# A Potential Combination Therapy of Berberine Hydrochloride With Antibiotics Against Multidrug-Resistant *Acinetobacter baumannii*

**DOI:** 10.3389/fcimb.2021.660431

**Published:** 2021-03-25

**Authors:** Xiaobo Li, Yanqing Song, Lina Wang, Guangbo Kang, Ping Wang, Huabing Yin, He Huang

**Affiliations:** ^1^ School of Chemical Engineering and Technology, Key Laboratory of Systems Bioengineering (Ministry of Education), Frontiers Science Center for Synthetic Biology, Tianjin University, Tianjin, China; ^2^ Tianjin Modern Innovative TCM Technology Co. Ltd., Tianjin, China; ^3^ School of Engineering, University of Glasgow, Glasgow, United Kingdom

**Keywords:** *Acinetobacter baumannii*, berberine hydrochloride, multidrug-resistance, antibiotic sensitization, synergistic effect, AdeABC

## Abstract

Multidrug-resistant (MDR) *Acinetobacter baumannii* strains can cause severe infections in intensive care units, and are rapidly developing resistance to the last-resort of existing antibiotics, posing a major global threat to health care system. Berberine hydrochloride (BBH), a kind of isoquinoline alkaloids extracted from Berberis and other plants, has been widely used as an antibacterial medicine for its reliable therapeutic efficiency. The *in vitro* synergistic effects of BBH with antibiotics against MDR *A. baumannii* were determined. BBH alone had weak antimicrobial activity (e.g., MIC≥256 mg/L) against MDR *A. baumannii*. However, it dramatically increased the susceptibility of MDR strains against antibiotics with FICI values <0.5, even reversed their resistance to antibiotics (e.g., tigecycline, sulbactam, meropenem and ciprofloxacin). *In vivo* study has suggested BBH with sulbactam had stronger antimicrobial efficiency than monotherapy in a neutropenic murine thigh infection model. The antibiotic-sensitizing mechanism of action of BBH was evaluated as well. BBH boosted *adeB* gene expression and bound to the AdeB transporter protein, resulting in low uptake of BBH, which may contribute to less extrusion of antibiotics by the AdeABC pump. Knockout of the *adeB* gene increased uptake of BBH and diminished the antibiotic sensitization and synergistic effects between antibiotics and BBH in MDR strains. Together, BBH effectively re-sensitizes this MDR pathogen to a range of antibiotics that have become barely effective due to antibiotic resistance, which indicates BBH may be a promising therapeutic adjuvant candidate to combat MDR *A. baumannii*.

## Introduction

*Acinetobacter baumannii* is a Gram-negative coccobacillus and considered the most virulent among all *Acinetobacter* species (Wong et al., 2017). As an opportunistic nosocomial pathogen, it can cause various community-acquired and hospital-acquired infections including pneumonia, urinary tract, lower gastrointestinal tract and surgical site infections, especially in intensive care units (Lee et al., 2017; Watkins, 2018). It is estimated that there is a high mortality rate when *A. baumannii* is present in community-acquired pneumonia cases. (Antunes et al., 2014). Due to its intrinsic and acquired antibiotic resistance (e.g. by mutations and gene transfer) along with improper use of antibiotics (Yoon et al., 2013), more and more multidrug-resistant (MDR) *A. baumannii* strains have been discovered. MDR *A. baumannii* has been ranked first on the critical priority list of MDR pathogens by the World Health Organization to guide research and development of antibiotics due to its greatest threat to human health (WHO, 2017).

Among the resistance mechanisms, multidrug efflux pumps play an important role. Three efflux systems AdeABC, AdeFGH and AdeIJK, which belong to the resistance-nodulation-cell division (RND) family, are associated with drug-resistance of MDR *A. baumannii* (Yoon et al., 2013; Nowak et al., 2016; Yoon et al., 2016). It has been reported the AdeABC pump contributes to resistance by extruding aminoglycosides, some β-lactams, fluoroquinolones, tetracyclines, tigecycline, macrolides, chloramphenicol, and trimethoprim *etc*. (Coyne et al., 2011; Chen et al., 2014). These MDR strains display resistance to almost all of today’s commonly prescribed antibiotics, leaving limited treatment options, which is becoming a major global issue and threatening the whole healthcare systems worldwide. For a long time, tigecycline and colistin have been considered last-resort drugs to treat this pathogen. However, they are now challenged by strong side effects and increasingly emerging resistance (Cai et al., 2012; He et al., 2019). Therefore, there is an urgent need to discover new antibacterial components targeting MDR *A. baumannii*. Considering the fact that discovery of antibiotic may take many years, researchers have now set their sights on natural sources (Gao et al., 2018).

**Figure 1:**
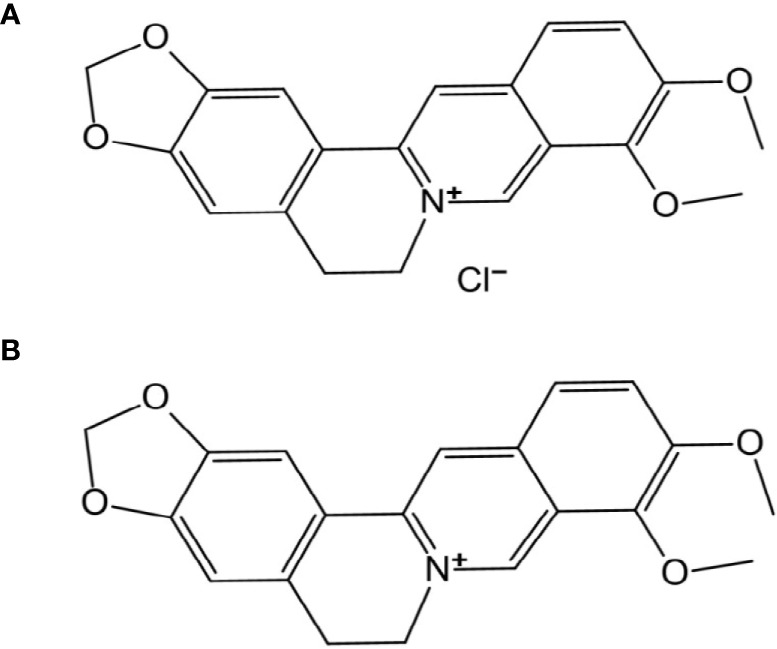
Chemical structure of **(A)** berberine hydrochloride and **(B)** berberine.

Berberine, a quaternary ammonium salt from the protoberberine group of isoquinoline alkaloids, can be found in many plants such as *Coptis chinensis* (a traditional Chinese herb, Huanglian), *Hydrastis canadensis*, *Berberis aristata*, *Phellodendron amurense*, *etc.* (Imenshahidi and Hosseinzadeh, 2016). Berberine hydrochloride (BBH) is the most common form of berberine. Their structures are shown in **Figure 1**. Both have been clinically used as an antibacterial medicine against gastroenteritis, abdominal pain and diarrhea in China for more than 2000 years (Tan et al., 2011; Gao et al., 2018). Relevant research has shown that berberine and its derivatives are efficient antibacterial agents against methicillin-resistant *Staphylococcus aureus* (MRSA) (Sun et al., 2019), *Acinetobacter baumannii* (Gao et al., 2018) and *Pseudomonas aeruginosa* (Morita et al., 2016). However, few studies have reported using BBH alone or in combination with antibiotics to treat MDR *A. baumannii*.

In this work, we evaluated the antimicrobial activity of BBH alone and in combination with several existing antibiotics against MDR *A. baumannii in vitro* and *in vivo*. Synergistic effects and antibiotic sensitization have been observed, and the mechanisms that BBH reserves resistance of MDR *A. baumannii* to the antibiotics have been investigated.

## Materials and Methods

### Bacterial Strains

Four strains of MDR *A. baumannii* (MDR-TJ, MDR-A, MDR-B and MDR-C) were isolated from the Second Hospital of Tianjin Medical University, China. Their drug-resistance profiles are listed in **Table S1**. In our previous study, we completed the whole genome sequence of MDR-TJ (GenBank accession number: NC_017847) and illustrated its mechanism of antibiotic resistance (Huang et al., 2012). The other two reference strains *A. baumannii* ATCC19606 and *Escherichia coli* ATCC 25922 were purchased from the American Type Culture Collection. All strains were stored in LB broth with 25% glycerol at -80°C.

### Antimicrobial Agents

Antimicrobial agents include berberine hydrochloride (BBH), amikacin, ciprofloxacin (Solarbio, Beijing, China), sulbactam (Aladdin, Shanghai, China), tigecycline (BioVision, San Francisco, USA), meropenem, and tetracycline (National Institutes for Food and Drug Control, Beijing, China). They were dissolved in sterile distilled water and stored at -20°C before the test. Unless otherwise noted, all antimicrobial powders have a purity greater than 98%. Efflux pump inhibitors carbonyl cyanide m-chlorophenylhydrazone (CCCP) and reserpine were purchased from Solarbio (Beijing, China), and phenylalanine-arginine β-naphthylamide (PAβN) from MedChemExpress (Shanghai, China).

### Determination of MICs

A two-fold broth microdilution method was used to determine the MICs of antimicrobials, following the guidance of the CLSI (M100) (Clinical and Laboratory Standards Institute, 2018). The MIC was defined as the lowest concentration of an antimicrobial agent that inhibits more than 90% bacterial growth from three independent tests, or as the lowest concentration with no visible bacterial growth (**Table S2**). A positive growth control, which contained bacterial suspensions in cation-adjusted Mueller-Hinton broth (CAMHB) without antimicrobials, and a negative control with broth only were included in a 96-well plate with a final volume of 200 μL. The plate was incubated for 20-24 h at 35°C in ambient air. *E. coli* ATCC 25922 was used as a quality control strain. The inhibition rate was calculated using the equation (1):

(1) $$\text{Inhibition rate}=\left(1-\frac{\text{OD}600\text{nm treatment}-\text{OD}600\text{nm negative control}}{\text{OD}600\text{nm positive control}-\text{OD}600\text{nm negative control}}\right)\times 100\%$$

Up to now, there are no CLSI susceptibility breakpoints for tigecycline and sulbactam against *A. baumannii*. Therefore, we used the CLSI criterion of ampicillin/sulbactam combination for sulbactam breakpoint and the FDA criterion for tigecycline breakpoint (*i.e.* MIC value ≤ 2 mg/L, susceptible; 4-8 mg/L, intermediate; ≥ 8 mg/L, resistant) (Navon-Venezia et al., 2007).

### Antimicrobial Combination Test by Checkerboard Assay

To determine the combinatory antimicrobial activities of antibiotics with BBH, the checkerboard assay was performed as previously described (Duarte et al., 2012). Briefly, a series of twofold dilutions of BBH and antibiotics were added in vertical and horizontal lines, respectively. The bacterial suspensions were added with a final concentration of about 5×10^5^ CFU/mL. The plate was incubated for 20-24 h at 35°C in ambient air. Interactions between antibiotics and BBH were determined according to the fractional inhibitory concentration index (FICI), which is interpreted as follows: FICI ≤ 0.5, synergy; 0.5≤FICI ≤ 4, no interaction; FICI>4, antagonism (Odds, 2003).

### Murine Thigh Infection Model

All animal procedures were performed at the Institute of Radiation Medicine Chinese and approved by the Animal Ethics and Welfare Committee of the Chinese Academy of Medical Science and Peking Union Medical College (Approval No. IRM-DWLL-2020106). The neutropenic mouse thigh infection model was utilized as previously described (Macvane et al., 2014; Barnes et al., 2019). Female ICR mice weighing 20 to 22 g were rendered transiently neutropenic *via* intraperitoneal injections of 150 and 100 mg/kg cyclophosphamide (National Institutes for Food and Drug Control, Beijing, China) at day 4 and day 1 prior to bacterial inoculation, respectively. Afterwards, 0.1 mL (~10^7^ CFU/mL) of bacterial suspension (MDR-TJ strain) was injected to both thighs of the mice under isoflurane anesthesia. After 2 h following infection, mice were randomly divided into 5 groups (n=5 mice/10 thighs), and the baseline group was euthanized to obtain the initial bacterial burden. Then Berberine hydrochloride (BBH) was administrated at 20 mg/kg q12h (*i.e.*, drugs were injected every 12 hours; 2 times total in a 24-h treatment period), sulbactam (SUL) at 400mg/kg q12h and combinations of BBH and SUL together as treatment regimens of monotherapy and combination therapy, respectively. All therapies were administered over a 24-h period by intraperitoneal injection of 0.2 mL doses each time, and total injection volume is 0.4 mL (0.2 mL BBH + 0.2 mL SUL) for combination therapy. Growth control animals were administered saline at the same volume and time interval. Subsequently, mice were euthanized, after which both thighs were excised aseptically, homogenized in 5 ml ice cold PBS (pH=7.2), and then plated on MHA plates by serial dilutions to determine bacterial burden expressed as Log_10_ CFU/thigh.

### Detection of the *adeABC* System and Relative Expression of the *adeB* Gene

Genomic DNA from *A. baumanii* strains was extracted using the Bacteria Genomic DNA Extraction Kit (Solarbio, Beijing, China). Genes *adeB*, *adeR*, *abeS*, and *16S rRNA* were detected by PCR. Expression of *adeB* was quantified by RT-PCR. In brief, *A. baumannii* isolates were grown in LB broth to reach mid-log phage with or without BBH, and then total RNA was extracted using the Trizol agent (Solarbio, Beijing, China). RT-PCR assays were performed using the SsoFast EvaGreen Supermix agent (Bio-Rad, USA) in the CFX96 Real-Time System (Bio-Rad, USA). The relative expression of the *adeB* gene was normalized against the housekeeping gene *16S rRNA* and calculated using the 2^−ΔΔCt^ method. *A. baumannii* ATCC 19606 was used as the reference strain, and RNase-free water was used as a negative control. All primers used in this study are listed in **Table S3**.

### Molecular Docking Study

To simulate how the antimicrobials bind to the AdeB transporter protein (AdeB) *in vitro*, theoretical molecular docking studies were performed. The crystal structure of AdeB (PDB code: 6OWS) (Su et al., 2019) was obtained from the RCSB Protein Data Bank, and the 3D structures of the antimicrobials were taken from the ZINC or PubChem database. AutoDock 4.2 was used to predict their bindings (Bikadi and Hazai, 2009). The results of docking computations were ranked by the binding energy, where the one with the highest energy was recorded. Finally, the protein-ligand docking models were visualized by PyMOL (http://www.pymol.org).

### *adeB* Gene Knockout

A marker-less gene deletion method was applied to delete the *adeB* gene (Amin et al., 2013). Briefly, a cross-over step was performed to select the MDR *A. baumannii* harboring the insertion plasmid Mo130-Tel^R^-*adeABC* (with a 1.9kb Up/Down fragment of *adeB*). It was followed by a second cross-over step to replace the *adeB* gene. Knockout was confirmed by the absence (i.e. undetectable) of the *adeB* gene amplification products as well as the presence of a 1.9kb Up/Down fragment amplification products by PCR using specific primers (**Table S3**).

### BBH Accumulation

BBH accumulation was measured as described previously with moderate changes (Wang et al., 2009). Briefly, bacteria were cultured into the exponential growth phase. They were centrifuged (8000×g, 2min), washed twice with 0.01M PBS (pH 7.2) and re-suspended in 1.5 mL PBS to OD_600nm_=1.0. BBH was added with a final concentration of 80 mg/L. 50 μM of an efflux pump inhibitor (*i.e.* CCCP, PAβN and reserpine) was added at an appropriate time (t = 60 min). Assays were performed in a 96-well black plate with a final volume of 100 μL per well. Fluorescence was measured every 5 min for 90 min in a microplate fluorometer (Thermo Scientific, USA) with an excitation wavelength of 355 nm and an emission wavelength of 538 nm.

### Statistical Analysis

All results were shown as mean ± standard deviation from three independent experiments with at least triplicate in each experiment. Unless otherwise noted, statistical analysis was carried out using one-way analysis of variance (ANOVA) by GraphPad Prism 8 (San Diego, CA, USA). P values < 0.05 indicate statistically significant difference.

## Results

### Antibiotic Effects of BBH Alone and in Combination With Others

We firstly examined the antimicrobial potential of BBH alone and its combined effects with antibiotics against *A. baumannii* (**Table 1**). BBH, as a single antimicrobial agent, showed a slight antibacterial activity, with a higher inhibitory effect on MDR-B (*i.e.* MIC at 256mg/L) than on the other three MDR stains and type stains (*i.e.* both MICs at 1024mg/L). The MDR isolates showed higher resistance to sulbactam, ciprofloxacin and meropenem compared with the type strain ATCC 19606. The variation in the sensitivities against different antibiotics of the three MDR strains may be due to different resistant genes harbored as they have different antibiotic susceptibility profiles (See **Table S1**).

**Table 1:** MICs profile and interactions between BBH and antibiotics.

Bacterial strains	Drugs	MICs (mg/L)			FICI _antibiotics_	FICI _BBH_	FICIs	Fold changes
		Alone	With BBH	BBH added^a^				
MDR-A	BBH	1024	–	–	–	–	–	–
	SUL	64 (R)	8 (I)	256	0.125	0.25	0.375	8^b^/4^c^
	**CIP**	**32 (R)**	**1 (S)**	**512**	**0.031**	**0.50**	**0.531**	**32/2**
	TGC	2 (S)	0.25 (S)	512	0.125	0.5	0.625	8/2
	MEM	128 (R)	8 (R)	256	0.063	0.25	0.313	16/4
MDR-B	BBH	256	–	–	–	–	–	–
	SUL	64 (R)	32 (R)	64	0.5	0.25	0.750	2/4
	CIP	32 (R)	4 (R)	256	0.125	1	1.125	8/1
	**TGC**	**4 (I)**	**2 (S)**	**16**	**0.5**	**0.063**	**0.563**	**2/16**
	MEM	64 (R)	8 (R)	128	0.125	0.5	0.625	8/2
MDR-C	BBH	1024	–	–	–	–	–	–
	**SUL**	**64 (R)**	**4 (S)**	**256**	**0.063**	**0.25**	**0.313**	**16/4**
	**CIP**	**32 (R)**	**1 (S)**	**256**	**0.031**	**0.25**	**0.281**	**32/4**
	TGC	1 (S)	0.25 (S)	512	0.25	0.5	0.75	4/2
	**MEM**	**128 (R)**	**2 (S)**	**256**	**0.016**	**0.25**	**0.266**	**64/4**
MDR-TJ	BBH	1024	–	–	–	–	–	–
	SUL	64 (R)	8 (I)	256	0.125	0.25	0.375	8/4
	**CIP**	**16 (R)**	**1 (S)**	**512**	**0.063**	**0.5**	**0.563**	**16/2**
	TGC	2 (S)	0.125(S)	512	0.063	0.5	0.563	16/2
	MEM	64 (R)	8 (R)	256	0.125	0.25	0.375	8/4
ATCC 19606	BBH	1024	–	–	–	–	–	–
	SUL	0.5 (S)	0.5 (S)	64	1	0.063	1.063	1/16
	CIP	1 (S)	0.25 (S)	256	0.25	0.25	0.5	4/4
	TGC	1 (S)	0.5 (S)	128	0.5	0.125	0.625	2/8
	MEM	0.5 (S)	0.125(S)	256	0.25	0.25	0.5	4/4

^a^BBH was added as the following concentrations: 512, 256, 128, 64, 32 and 16 mg/L, where the concentration was recorded to obtain lowest FICI value. ^b^fold changes of MICs of antibiotics without or with BBH; ^c^fold changes of MICs of BBH without or with antibiotics.

BBH, berberine hydrochloride; SUL, sulbactam; TGC: tigecycline; CIP, ciprofloxacin; MEM: meropenem. S, susceptible; R, resistant; I, intermediate.

Bold marks indicate antibiotic resensitization.

As shown in **Table 1**, BBH in combination with antibiotics showed a more significant antimicrobial potential. Synergistic and antagonistic interactions between BBH and the tested antibiotics were indicated by FICIs. Several combinations showed 0.5<FICIs<1, suggesting a weak synergistic interaction between the BBH and antibiotics. Synergistic effects (FICI<0.5) were observed in the combinations of BBH/sulbactam and BBH/meropenem for the MDR strains. Furthermore, the addition of BBH reduced the MIC values of the majority of tested antibiotic-pathogen pairs and made the MDR strains re-sensitive to several antibiotics (Indicated by bold marks in **Table 1**). For example, the MIC of ciprofloxacin against MDR-A decreased from 32 to 1 mg/L, and the MIC of sulbactam and meropenem against MDR-C decreased from 64 to 4 mg/L and from 128 to 2 mg/L, respectively. This phenomenon demonstrates that BBH has the potential to reverse antibiotic resistance or enhance the susceptibility of *A. baumannii* to those no-longer-effective antibiotics. Therefore, BBH could be an effective adjuvant to bring those off-the-treatment-list antibiotics (*e.g.* sulbactam and ciprofloxacin) back to use for treating MDR *A. baumannii* infections.

### Murine Thigh Infection Model of MDR *A. baumannii*

**Figure 2:**
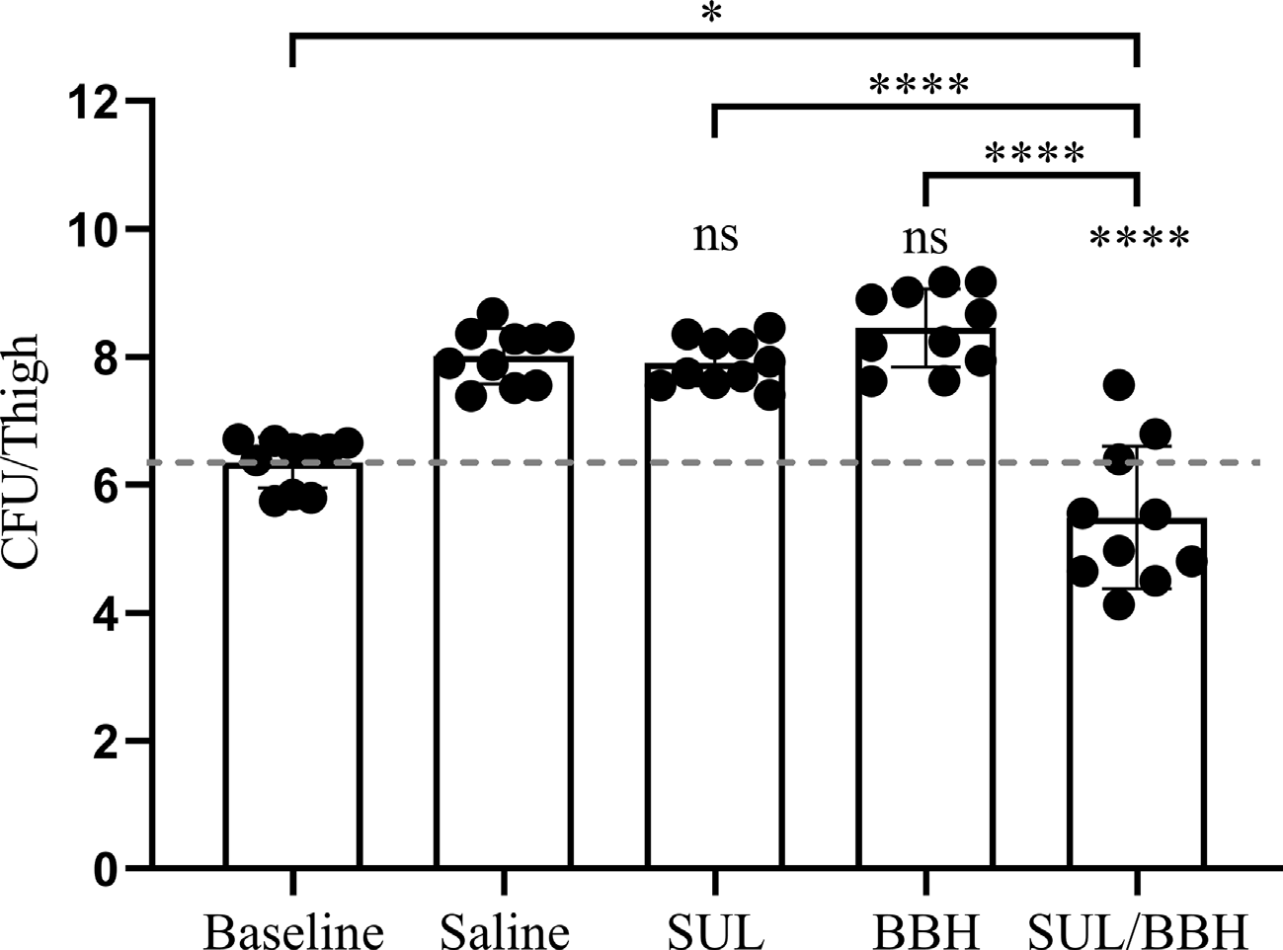
Efficiency of SUL/BBH combination therapy against MDR-TJ in murine thigh infection model (n = 5 mice, 10 thighs). The data were obtained from one single test and statistical analysis was carried out using one-way analysis of variance (ANOVA) with Tukey’s test by GraphPad Prism 8. ns, no significance; *P < 0.05; ****P < 0.0001.

The MDR *A. baumannii* strain MDR-TJ was used to establish a murine thigh infection model for its board spectrum of drug-resistance profiles (See **Table S1**). This strain demonstrates resistant to sulbactam *in vitro* with an MIC of 64 mg/L, however, susceptibility is changed in the presence of BBH with a MIC of 8 mg/L (**Table 1**). The toxicity of BBH (pH=7.2) was evaluated prior to *in vivo* combination therapy tests, and the dose of 20 mg/kg administrated intraperitoneally was safe with in a 24-h period (See **Figure S1**). Monotherapy of SUL and BBH individually every 12 h with doses of 400 mg/kg and 20 mg/kg couldn’t inhibit bacteria growth, resulting increment of 1.56 and 2.11 Log_10_ CFU/thigh in 24h treatment compared with baseline value (**Figure 2**). Whereas, BBH can effectively boost the efficiency of SUL *in vivo* against MDR-TJ strain, making in a 2.53 Log_10_ CFU/thigh reduction, even shows a bactericidal action (-0.86 Log_10_ CFU/thigh reduction compared with baseline value).

### Detection of the *adeABC* System and *adeB* Gene Expression

Given that BBH reduced more than 4-fold MICs of antibiotics in most combinations, it presents a similar effect of efflux pump inhibitors (Coyne et al., 2011). Therefore, we evaluated whether *A. baumannii* has the most prevalent efflux pump AdeABC system (multidrug transporter protein gene *adeB* and its two-component regulatory system genes *adeR* and *adeS*) (Coyne et al., 2011; Yoon et al., 2015). In all three strains, we detected the *adeB* and *adeR-adeS* genes by PCR (**Figure S2**) and the sequences of amplification products were highly identical with the target genes (≥95%). This proves that all strains in this study have the *adeABC* efflux pump system, including the sensitive strain ATCC19606.

**Figure 3:**
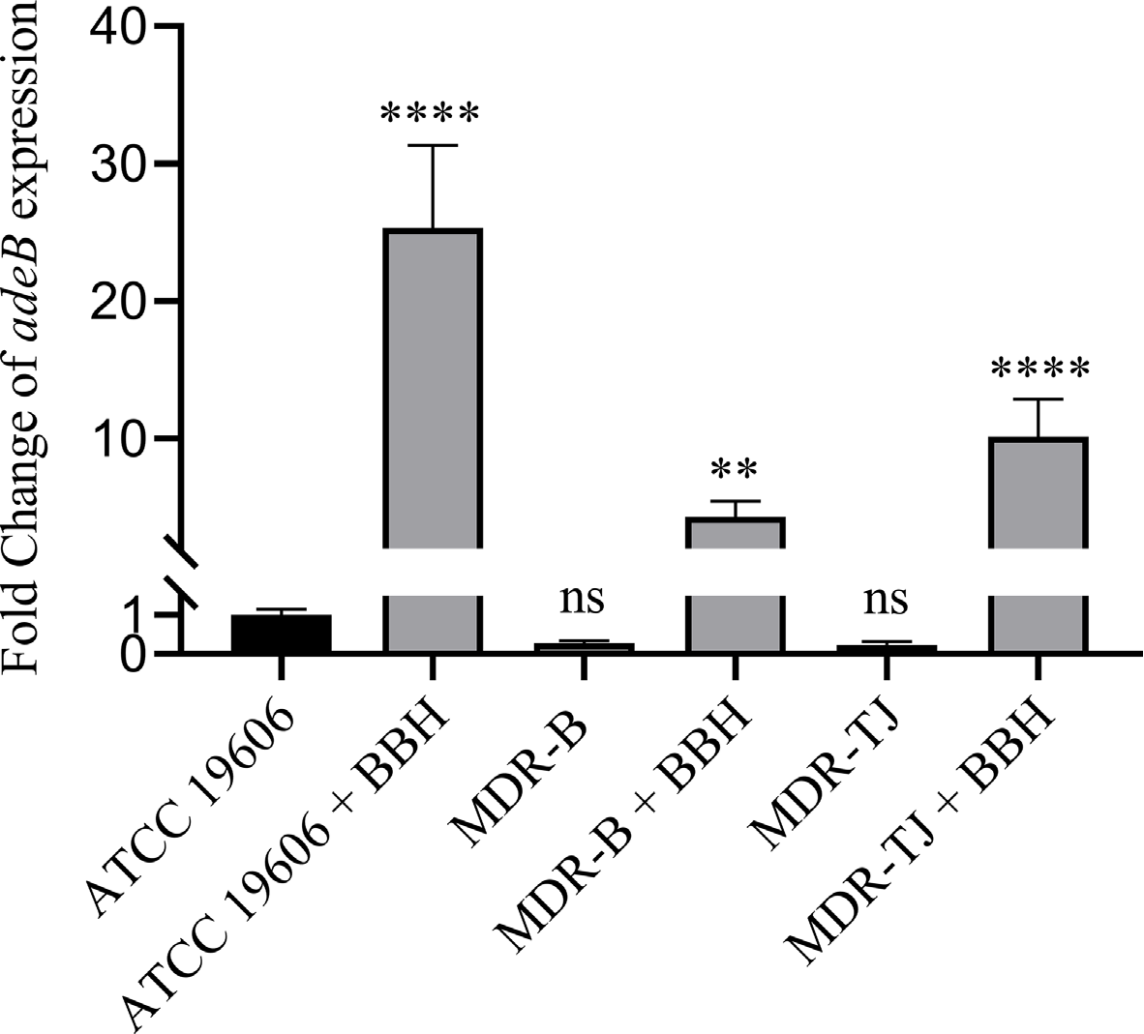
Quantification of *adeB* expression normalized with *A. baumannii* ATCC 19606. BBH was added at half MIC corresponding to each stain, which is 128 mg/L for MDR-B and 512 mg/L for MDR-TJ and ATCC 19606, respectively. Statistical analysis was carried out using one-way analysis of variance (ANOVA) with Tukey’s test by GraphPad Prism 8. ns, no significance; **P < 0.01; ****P < 0.0001.

The relative expressions of the *adeB* gene in the three strains with or without BBH are shown in **Figure 3**. Without BBH, there was no difference in *adeB* expression between the MDR strains and the ATCC 19606 strain. Following the addition of BBH, the expression of the *adeB* gene increased by 25.3, 4.3, 10.2 folds for ATCC 19606, MDR-B and MDR-TJ, respectively. The expression of multidrug pumps is typically subjected to induction by their substrates (Coyne et al., 2011). Thus, this phenomenon indicates that BBH is more likely to be recognized by the bacteria as a substrate for the AdeABC system rather than an inhibitor.

### Molecular Docking Study

**Figure 4:**
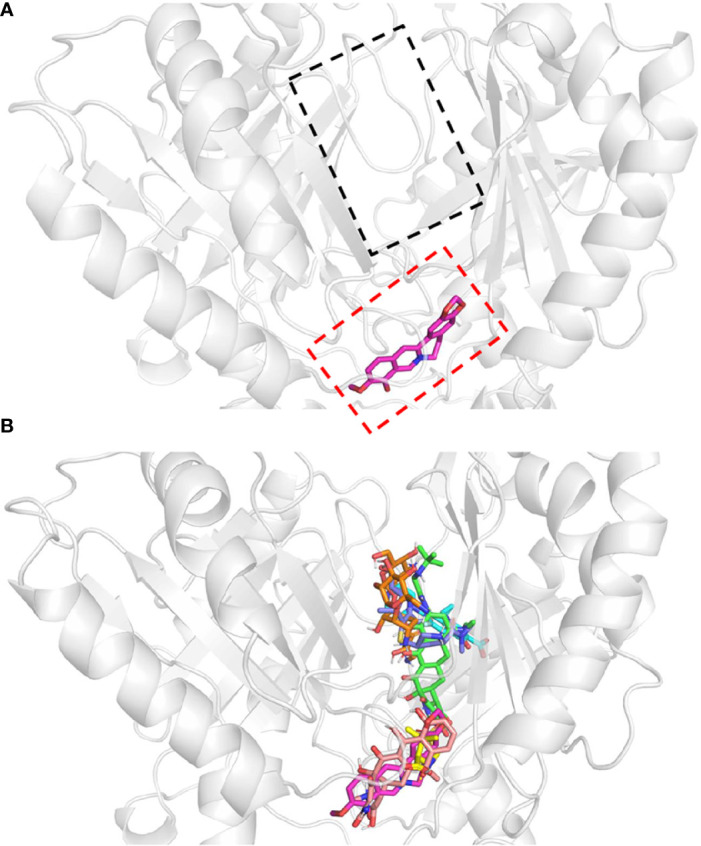
Protein-ligand docking models. Docking structure of **(A)** berberine, and **(B)** antibiotics. All antibiotics are close to the proximal multidrug binding site (F-loop, red frame; G-loop, black frame) of AdeB. Berberine, pink; tigecycline, green; ciprofloxacin, cyan; sulbactam, yellow; tetracycline, light salmon; meropenem, dark blue; amikacin, orange.

To examine whether BBH could be a competitive substrate of AdeB protein, protein-ligand docking models were built (**Figure 4**), and their binding energies were computed (**Table 2**). Berberine was used as a substitute for BBH due to their identical 3D structure when dissolved. Six antibiotics (*i.e.* tigecycline, ciprofloxacin, sulbactam, tetracycline, meropenem, and amikacin) were also simulated, where tigecycline, ciprofloxacin, tetracycline and amikacin were proved as substrates of AdeB protein (Coyne et al., 2011).

**Table 2:** Binding energy of protein-ligand docking models.

Antimicrobials	Residues^a^ (form hydrogen bonds)	Binding energy (kcal/mol)
Berberine	–	-7.42
SUL	–	-5.79
TGC	Thr-668/Glu-665/Leu-666	-7.34
AMK	Trp-610/Ser-613/Thr-668	-6.76
CIP	–	-6.45
MEM	Trp-610/Ser-613	-5.98
TET	Glu-665	-10.05

Residues^a^, Only the residues of AdeB that play a significant role in the pump efflux function were considered, which are supposed to be Trp-610, Phe-612, Ser-613, Ala-615, Pro-661, Ala-662, Ile-663, Asp-664, Glu-665, Leu-666, Thr-668.

SUL, sulbactam; TGC, tigecycline; AMK, amikacin; CIP, ciprofloxacin; MEM, meropenem; TET, tetracycline.

The multidrug transporter protein AdeB has three multidrug binding sites: the periplasmic cleft entrance, proximal multidrug binding site (F-loop and G-loop) and hydrophobic patch (Su et al., 2019). The F-loop and G-loop contain many multidrug binding sites (e.g. Trp-610, Phe-612, Ser-613, Ala-615, Pro-661, Ala-662, Ile-663, Asp-664, Glu-665, Leu-666, Thr-668), which can be found in other multidrug pumps, such as Cus A, AcrB, and MtrD pumps (Su et al., 2019). Therefore, these sites were selected as the docking sites. The docking model showed that all of these drugs were located in the proximal multidrug binding sites of AdeB (**Figure 3**). Binding energy calculations showed that berberine had higher binding energy (*c.a.* -7.42 kcal/mol) to AdeB than all the other antibiotics except tetracycline, which indicates that BBH is a positive substrate of the multidrug transporter protein AdeB (**Table 2**).

### *adeB* Gene Deletion and MIC Reduction

The deletion of the *adeB* gene was confirmed by comparing PCR amplification products between the parental strains (MDR-B and MDR-TJ) and the mutant strains (△*adeB* MDR-B and △*adeB* MDR-TJ) (**Figure S3**). The parental strains generated the *adeB* gene amplification products and about 5.2kb Up/Down-fragment amplification products, whereas the mutants lacked the *adeB* gene products and generated about 1.9kb Up/Down-fragment products. These results showed that *adeB* knockout was successful in △*adeB* MDR-B and △*adeB* MDR-TJ mutants. Furthermore, sequencing results were identical to the target sequences.

To verify the mechanism that BBH is a competitive substrate of the AdeB protein, the △*adeB* MDR-TJ was used to determine the MICs of the selected antibiotics with and without BBH (**Table 3**). The assumption is that synergistic effects between BBH and antibiotics would diminish or antibiotic sensitization would disappear in *adeB* deletion strains. As is shown in **Table 3**, compared to the parental MDR-TJ strain, △*adeB* MDR-TJ showed reduced MICs of sulbactam, tigecycline and ciprofloxacin, indicating that the lack of expelling these antibiotics by the AdeB protein can enhance the killing effect of the antibiotics. It should be noted that there was no difference in the MIC of BBH alone between the parental MDR-TJ strain and the △*adeB* MDR-TJ strain. This indicates that other efflux systems might also play an important role in the extrusion of BBH. For the △*adeB* MDR-TJ strain, the addition of BBH only induced a 2-fold decrease of the MICs for all antibiotics but sulbactam (no change with BBH) (**Table 3**). In contrast, the addition of BBH induced more than an 8-fold decrease of the MICs for the corresponding antibiotics in the parental MDR-TJ strain (**Table 1**). Furthermore, while the presence of BBH reversed the MDR-TJ strain susceptibilities to sulbactam and ciprofloxacin, the addition of BBH did not change the susceptibility of the △*adeB* MDR-TJ strain to any of the antibiotics tested. What’s more, the FICIs were more than 0.5, indicating no synergistic effects between antibiotics and BBH observed in △*adeB* MDR-TJ strain. If there is any other reason, in the deletion strain of MDR-TJ, the MIC of antibiotic with BBH will decrease. Whereas, as shown in **Table 3**, only one to two-fold MIC reduction was observed. Therefore, these data could be proved that the main target of BBH is AdeABC. Taken together, these data suggest that a key role of BBH is to compete against antibiotics for the binding sites of AdeB, which increased antibiotic susceptibility of MDR *A. baumannii*.

**Table 3:** MICs in △*adeB* MDR-TJ.

Drugs	MICs (mg/L)			FICIs	Fold changes of antibiotics	
	Alone	With BBH	BBH added^a^		MIC_△_ *_adeB_*_MDR-TJ_/MIC_△_ *_adeB_*_MDR-TJ+BBH_	MIC_MDR-TJ_/MIC_△_ *_adeB_*_MDR-TJ_
BBH	1024	–	–	–	–	1
SUL	32 (R)	32 (R)	64	1.063	1	2
TGC	0.25 (S)	0.125(S)	512	1	2	8
CIP	8 (R)	4 (R)	512	1	2	2
MEM	64 (R)	32 (R)	128	0.625	2	1

a BBH was added as the following concentrations: 512, 256, 128 and 64 mg/L, where the concentration was recorded to obtain lowest FICI value.

BBH, berberine hydrochloride; SUL, sulbactam; TGC, tigecycline; CIP, ciprofloxacin; MEM, meropenem. S, susceptible; R, resistant; I, intermediate.

### BBH Accumulation Study

**Figure 5:**
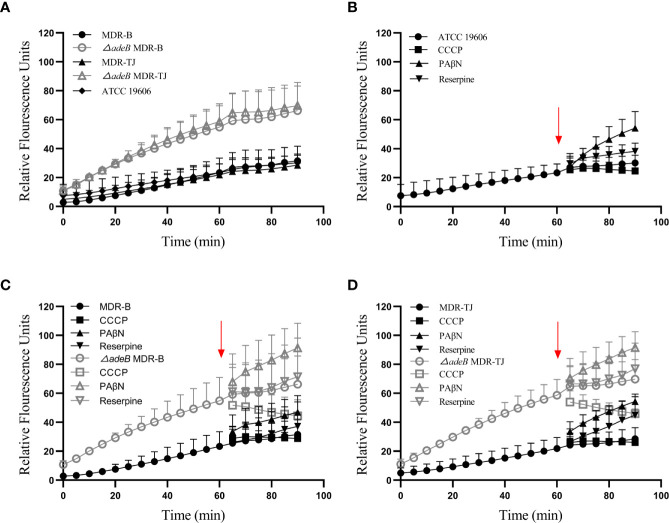
BBH accumulation in *A. baumannii* strains. The amount of BBH was determined as relative fluorescence units. **(A)** BBH uptake by *A. baumannii* strains in 90 min. BBH uptake with pump inhibitors CCCP, PAβN, and reserpine in **(B)** ATCC 19606 strain, **(C)** MDR-B and △*adeB* MDR-B strains, and **(D)** MDR-TJ and △*adeB* MDR-TJ strains. Red arrows indicate the pump inhibitors (50 µM) were added at 60 min. Grey letters indicate the inhibitors were added to the *adeB* knockout strains.

The interior of bacteria is always negatively charged relative to the external environment. Therefore, BBH, as a cationic quaternary ammonium salt, can be easily retained within cells by membrane potential (Severina et al., 2001). It is reported that BBH, likely ethidium bromide, may bind to DNA or cytoplasmic membrane, resulting in increased fluorescence (Wang et al., 2009). Thereby, we utilized this phenomenon to estimate the uptake of BBH within bacteria, which were measured as relative fluorescence units (RFUs). As shown in **Figure 5A**, the uptake of BBH reached a stationary stage at about 60 min in the MDR-B, MDR-TJ and ATCC 19606 strains, suggesting that BBH may be extruded by bacteria through efflux pumps in general. For the △*adeB* MDR-B and △*adeB* MDR-TJ strains that did not produce any AdeB protein, significantly higher accumulation of BBH was observed in comparison with their parental strains, suggesting that part of BBH can be extruded by AdeB.

To further understand the mechanism of action of BBH, efflux pump inhibitors CCCP, PAβN and reserpine were used to evaluate BBH accumulation (**Figures 5B–D**). PAβN mainly causes membrane permeabilization (Li et al., 2015), and it increased BBH accumulation in all the strains. Reserpine is a blocker of P-glycoprotein (Li et al., 2015), and caused a marginal decline of antibiotic resistance in MDR *A. baumannii* as a non-specific pump inhibitor (Pule et al., 2016). The addition of reserpine only slightly increased BBH accumulation, indicating other efflux pumps may contribute to BBH extrusion. CCCP as a proton uncoupler can inhibit the energy of efflux pumps by reducing transmembrane potential (Pule et al., 2016; Shi et al., 2018). It would increase BBH accumulation in this regard when BBH acts as a competitive substrate. However, our results showed a reduction of BBH in bacteria after the addition of CCCP, suggesting the occurrence of BBH leakage (**Figures 5C, D**). A plausible reason is that CCCP may inhibit the proton motive force on the outer membrane responsible for the penetration of BBH into the interior of bacteria (Severina et al., 2001).

## Discussion

Nowadays, antibiotic resistance of *A. baumannii* has progressively increased, reducing the range of clinically available treatment options. The increasing cases of MDR *A. baumannii* accompanied by a scarcity of new antibiotics have led to monotherapy ineffective against this pathogen. It’s been reported transmission of MRD microorganisms accompanying with fecal microbiota transplantation (FMT) could lead to adverse infections, even causing fatal cases (DeFilipp et al., 2019). Combination therapy has been considered an effective approach to combat MDR *A. baumannii* (Worthington and Melander, 2013). It may offer a synergism even when MDR strains are not susceptible to the single antibiotics, but occasionally antagonism may be observed (Durante-Mangoni et al., 2014). In addition, in the process of combination therapy for infections with MDR or extensively drug-resistant (XDR) strains, colistin (polymyxin E) serves as one part of the mixtures most of the time, however, side effects including nephrotoxicity limit its clinical utility (Viehman et al., 2014; Wang et al., 2019). Tigecycline or tigecycline-based therapy has excellent activity against *A. baumannii*, but it has been associated with higher treatment failure rates than non-tigecycline therapy according to a study of 238 patients with carbapenem-resistant *A. baumannii* pneumonia (Liang et al., 2018). All in all, safe antimicrobial agents with excellent antibacterial activity should be discovered to address this dilemma. However, only five first-in-class antibiotics have been approved in the past two decades, none of which targets Gram-negative bacteria (Butler and Paterson, 2020). Although there are some β-lactamase/β-lactam inhibitor (BLI) combinations have been approved to against Gram-negative bacteria, the β-lactamases have been approved previously (Butler and Paterson, 2020).

Recently, researchers have begun to revitalize antibiotic development from natural herb compounds or clinically proven drugs against Gram-negative bacteria. For instance, halicin (Stokes et al., 2020) and SCH-79797 (Martin et al., 2020) were discovered to kill Gram-negative bacteria with a unique mechanism, which can provide novel promising antibiotic candidates. Similarly, we set our sights on Traditional Chinese Medicine for its time-honored clinical safety evaluation and effective treatment. Here, we discovered that BBH is a potential antimicrobial adjuvant against MDR A. baumannii when combined with antibiotics. We may conclude that BBH can attenuate β-lactamase or β-lactam inhibitor (meropenem and sulbactam) resistance, quinolone (ciprofloxacin) resistance and glycylcycline (tigecycline) resistance against MDR *A. baumannii* rather than aminoglycoside resistance of *A. baumannii*. For instance, the MIC values of amikacin (an aminoglycoside antibiotic) of MDR-B and MDR-TJ were >1024 mg/L with or without berberine hydrochloride (data did not show in this paper), which represented similar results with previous study (Morita et al., 2016). What’s more, combination therapy of BBH and SUL against MDR-TJ strain in the murine thigh infection model revealed stronger antimicrobial or bactericidal efficiency than monotherapy. Admittedly, the *in vivo* test was limited for we mainly focused on the combination of SUL/BBH. In future, a series of work may be carried out to investigate more combination therapies between different antibiotics and berberine hydrochloride against different *A. baumannii* isolates, accompanied with collecting data of pharmacokinetic/pharmacodynamic (PK/PD), which may provide more valuable data for clinical trials.

Genomic analysis based on whole-genome sequencing is a significant way to reveal the mechanism of antibiotic resistance in *A. baumannii*. Our previous research indicated *A. baumannii* strain MDR-TJ could harbor many resistance-associated genes inferring different resistance mechanisms, such as enzyme-mediated degradation, alteration of target sites, and efflux pumps (Huang et al., 2012). Among them, efflux pumps contribute to the extrusion of most antibiotics. In MDR-TJ, a specific efflux pump AdeABC belonging to the RND family has been characterized, which mediates the resistance to a broad spectrum of antibiotics, whereas AdeIJK, another efflux pump identified, is considered to be related to the inherent resistance of *A. baumannii* (Coyne et al., 2011). Therefore, we focused the target of BBH against MDR *A. baumannii* on pump AdeABC based on the identification of drug-resistance genes in MDR-TJ and the fact that BBH can resume antibiotic sensitivity. Previous studies indicated that cationic antibacterial agents (*e.g.* berberine) are ideal substrates of multidrug pumps and actively extruded by multidrug pumps in MDR strains (Hsieh et al., 1998; Morita et al., 1998; Stermitz et al., 2000; Severina et al., 2001; Tegos et al., 2002). Our results were consistent with those findings. BBH uptake reached a stationary stage at about 60 min, indicating a balance of accumulation and extrusion by membrane potential and multidrug pump, respectively. The significant increases in the amount of BBH accumulated in the △*adeB* strains or in the presence of pump inhibitor PAβN validated the competitive role of BBH in the efflux-mediated extrusion of antibiotics (**Figure 5**).

Some studies showed that the antibacterial activity of berberine and its derivatives is attributed to their role as a pump inhibitor (Morita et al., 2016; Pule et al., 2016). Our study demonstrated that BBH is more likely a pump competitor to resume antibiotic sensitivity than an inhibitor in the treatment of *A. baumannii*, because it significantly boosted the expression of pump gene *adeB* and had a higher affinity toward AdeB than antibiotics (**Figure 3** and **Table 2**). In addition, the results that antibiotic-sensitizing activity and synergistic effects diminished in △*adeB* strain (**Table 3**) confirmed BBH could facilitate antibiotic efficacy by competing against antibiotics for the AdeB sites. However, the MICs of BBH did not decrease in the △*adeB* strains, and they changed slightly with pump inhibitors (**Table S4**), which implies that AdeB may not be the only efflux pump for BBH and other pumps may also contribute to BBH extrusion. Despite the poor absorption and bioavailability of BBH (Imenshahidi and Hosseinzadeh, 2016) as a single antimicrobial, it still has accumulated numerous reliable therapeutic data, and novel approach, like nanoparticulate delivery system (Tan et al., 2011), has been developed to improve its bioavailability. In contrast, for most efflux pump inhibitors (*i.e.* CCCP and PAβN), the strong side effects and toxicity limit their clinical applications (Li et al., 2015). Last but not least, berberine and berberine analogs which may have similar efficacy against MDR *A. baumannii* are worthy of further research and development *in vitro* and *in vivo* to alleviate imminent threats to human health posed by the continued emergence of multidrug-resistant bacteria.

## Conclusions

**Figure 6:**
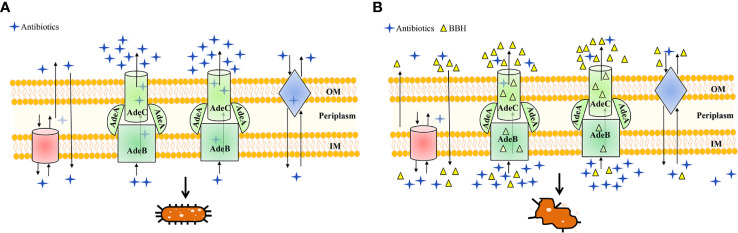
Schematic diagram highlighting the antibiotic efflux mechanisms with **(A)** BBH absent or **(B)** present. MDR *A. baumannii* may harbor abundant pump systems. For instance, AdeABC pump contributes to resistance by extruding a wide range of antibiotics into the extracellular environment, which causes a lower dose of antibiotics to accumulate within bacteria and leads to multidrug resistance and concomitant cell growth, as illustrated in **(A)**. However, in the presence of BBH **(B)**, multidrug resistance of antibiotics can be reversed for it could occupy the binding sites of AdeABC and other efflux proteins as a positive pump competitor. This way both the penetration of antibiotics from the outer membrane (OM) and accumulation inside the inner membrane (IM) are maintained, leading to the killing of bacteria.

Collectively, our findings have demonstrated that BBH is a positive substrate of AdeB and possibly other efflux pumps, which may be responsible for the synergistic effects of BBH with antibiotics and antibiotic-sensitizing activity in MDR *A. baumannii* (**Figure 6**). *In vivo* study has suggested that BBH can dramatically boost the antimicrobial efficiency of sulbactam against MDR-TJ strain, which may provide a novel combination therapy to combat MDR *A. baumannii* infection. Meanwhile, despite of the substantial and clear results *in vitro* tests, more *in vivo* studies are needed to carry out in the future to reinforce the clinical effectiveness of the combination therapies.

## Data Availability Statement

The raw data supporting the conclusions of this article will be made available by the authors, without undue reservation.

## Ethics Statement

The animal study was reviewed and approved by The Chinese Academy of Medical Science and Peking Union Medical College (Approval No. IRM-DWLL-2020106).

## Author Contributions

HH, HY, PW and XL conceived and designed this experiment. XL, YS, LW and GK performed the experiments. XL, GK and YS analyzed the data. XL and YS wrote the draft manuscript. All authors contributed to the article and approved the submitted version.

## Funding

This work was supported by grants from National Key Research and Development Project (No. 2019YFA0905600), Major State Basic Research Development Program of Natural Science Foundation of Shandong Province in China (No. ZR2020ZD11) and Science and Technology Program of Tianjin, China (No. 19YFSLQY00110). We thank partial support of NERC (NE/S008721/1).

## Conflict of Interest

Authors XL and PW were employed by Tianjin Modern Innovative TCM Technology Co. Ltd.

The remaining authors declare that the research was conducted in the absence of any commercial or financial relationships that could be construed as a potential conflict of interest.
