# Supplementary material for: A Potential Combination Therapy of Berberine Hydrochloride With Antibiotics Against Multidrug-Resistant Acinetobacter baumannii
Source: Front Cell Infect Microbiol. 2021 Mar 25;11:660431. doi: 10.3389/fcimb.2021.660431 (PMC8027359; doi:10.3389/fcimb.2021.660431)
Supplement: Supplementary file 1 [file DataSheet_1.docx]

Supplementary Material

# Supplementary Data

## Antibiotic susceptibility profiles of MDR A. baumannii

**Table S1.** Antibiotic susceptibility profiles of MDR *A. baumannii*

| Antibiotic classes | Antibiotics | MIC (mg/L) | | | | | | | |
| --- | --- | --- | --- | --- | --- | --- | --- | --- | --- |
|  |  | MDR-A | | MDR-B | | MDR-C | | MDR-TJ | |
| Quinolones | levofloxacin | - | - | 16 | R | 4 | M | ＞8 | R |
|  | ciprofloxacin | ＞4 | R | 32 | R | ＞4 | R | ＞4 | R |
|  | ofloxacin | - | - | 16 | R | - | - | - | - |
|  | moxifloxacin | - | - | ＞8 | R | - | - | - | - |
| Cephalosporins | ceftazidime | ＞64 |  | 256 | R | ＞64 | R | ＞64 | R |
|  | cefotaxime | - | - | ＞1024 | R | - | - | - | - |
|  | cefoperazone | - | - | ＞1024 | R | - | - | - | - |
|  | ceftriaxone | - | - | ＞1024 | R | - | - | - | - |
|  | cefoperazone/  sulbactam | ＞64 |  | 64 | R | ＞64 |  | ＞64 | R |
| Aminoglycosides | amikacin | - | - | ＞1024 | R | - | - | ＞64 | R |
|  | streptomycin | - | - | ＞16 | R | - | - | - | - |
|  | gentamicin | - | - | ＞1024 | R | - | - | - | - |
|  | tobramycin | ＞16 | R | - | - | ＞16 | R | ＞16 | R |
| Carbapenems | imipenem | ＞16 | R | ＞16 | R | ＞16 | R | ＞16 | R |
|  | meropenem | ＞16 | R | ＞16 | R | ＞16 | R | ＞16 | R |
| Penicillins | piperacillin/  tazobactam | ＞128 | R | 512 | R | ＞128 | R | ＞128 | R |
| Tetracyclines | tetracycline | - | - | ＞16 | R | - | - | - | - |
|  | minocycline | - | - | - | - | - | - | ＞16 | R |
|  | doxycycline | - | - | - | - | ＞16 | R | ＞16 | R |
| Sulfonamides | sulfisoxazole | - | - | ＞512 | R | - | - | - | - |
|  | trimethoprim/  sulfamethoxazole | - | - | - | - | ＞320 | R | ＞320 | R |
| Polymyxins | colistin | - | - | - | - | - | - | 4 | R |

Note: R, resistant; S, susceptible; I, intermediate.

## MIC values between two methods: inhibition more than 90% and no visible growth

**Table S2**. MIC values based on two methods

| Antimicrobial | MICs(mg/L) | | |
| --- | --- | --- | --- |
|  | *A. baumannii*  MDR-B | *A. baumannii*  MDR-TJ | *A. baumannii*  ATCC 19606 |
| BBH | 256**^a^**/256**^b^** | 1024/1024 | 1024/1024 |
| SUL | 64/64 (R) | 64/64 (R) | 0.5/0.5 (S) |
| TGC | 4/4 (M) | 2/2 (S) | 1/1 (S) |
| AMK | >2048/>2048 (R) | >2048/>2048 (R) | 32/32 (M) |
| CIP | 32/32 (R) | 16/32 (R) | 1/1 (S) |
| MEM | 64/64 (R) | 64/64 (R) | 0.5/1 (S) |
| TET | >2048/>2048 (R) | >2048/>2048 (R) | 4/4 (S) |

a: MIC value based on more than 90% inhibition; b: MIC value based on no visible growth from three independent tests.

Both methods showed identical results of susceptibility, however, method (a) was more efficient to determine the MIC value when BBH was tested because the bright yellow color of BBH makes it difficult to observe cell growth.

## Primers used in this study

**Table S3.** Primers used in this study for PCR and RT-qPCR

| Gene | Primer sequence (5’→3’) | Length (bp) | Note |
| --- | --- | --- | --- |
| *adeB* | F:GAAGATCAAGGTTGGTTCATGAC | 815 | *adeABC* detected |
|  | R: ATTTCACGCATTGCTTCACC |  |  |
| *adeR* | F: GAAGGCATGAGTGTTATTCGG | 526 | *adeABC* detected |
|  | R: GGCTATCTACGGTTCGCTCTA |  |  |
| *adeS* | F: GCCAATTAACTTCTTAGCCG | 746 | *adeABC* detected |
|  | R: TGCCTTTCAGTGCCACAAT |  |  |
| *16S rRNA* | F: CTACCAAGGCGACGATCTGT | 881 | *adeABC* detected |
|  | R: GTTCCCATCCGAAATGCTG |  |  |
| *adeB* | F: CGCAAGTTGTACGTCAGCA | 170 | qRT-PCR |
|  | R: AACCTTCCCTACACCTTCGA |  |  |
| *16S rRNA* | F: AACGCGAAGAACCTTACCTG | 133 | qRT-PCR |
|  | R: GCGGGACTTAACCCAACAT |  |  |
| Up-Down fragment | F: CCGCAATCGGTAAATTTTAGTG | 1968/5278^a^ | *adeB*  knockout |
|  | R:GATATTAGGTCCAATCGACCATGC |  |  |
| *adeB* | F: TGCGACAACAGATACCTCCG | 966 | *adeB*  knockout |
|  | R: GCAAGCACCATACCGAACAT |  |  |

Note: F: forward; R: reverse.

a: The length of PCR product of Up-Down fragment is 1968bp for the mutant strain and 5278bp for the parental strain, respectively.

## MICs of BBH

**Table S4.** MICs of BBH in △*adeB* strains or with pump inhibitors

| Strains | MICs of BBH (mg/L) | | | | |
| --- | --- | --- | --- | --- | --- |
|  | - | △*adeB* | + CCCP^a^ | + PAβN^a^ | +reserpine^a^ |
| MDR-B | 256 | 1024 | 64 | 1024 | >1024 |
| MDR-TJ | 1024 | 1024 | 64 | 512 | 512 |
| ATCC 19606 | 1024 | - | 32 | 1024 | >1024 |

a: The final concentration of efflux pump inhibitors CCCP, PAβN and reserpine was 50 μM. To be noted, bacteria were inhibited less than 50% at this concentration.

## Toxicity of BBH


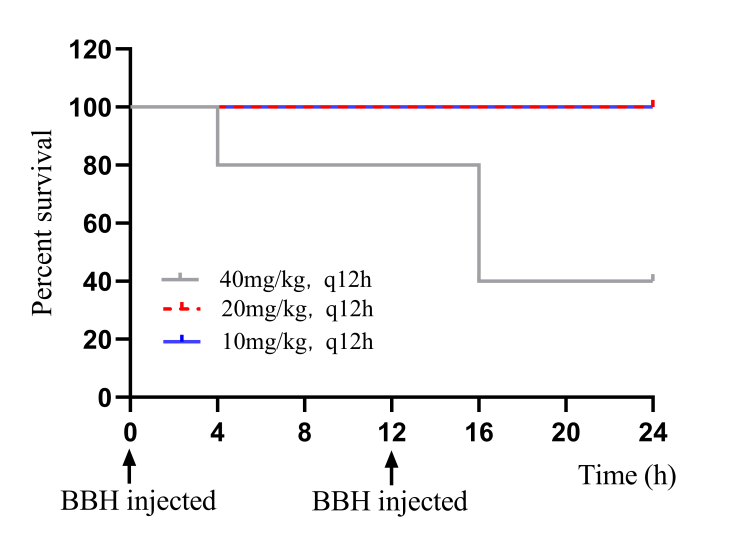


**Figure S1.** Survival curve of BBH to mice (n=5). BBH (pH=7.2) was administrated intraperitoneally with doses of 10 mg/kg, 20 mg/kg and 40 mg/kg every 12 h with a period of 24 hours, respectively. It showed that only 40 mg/kg BBH presented toxic to mice, giving a 40% survival rate after 24h treatment.

## Gel pictures in this study


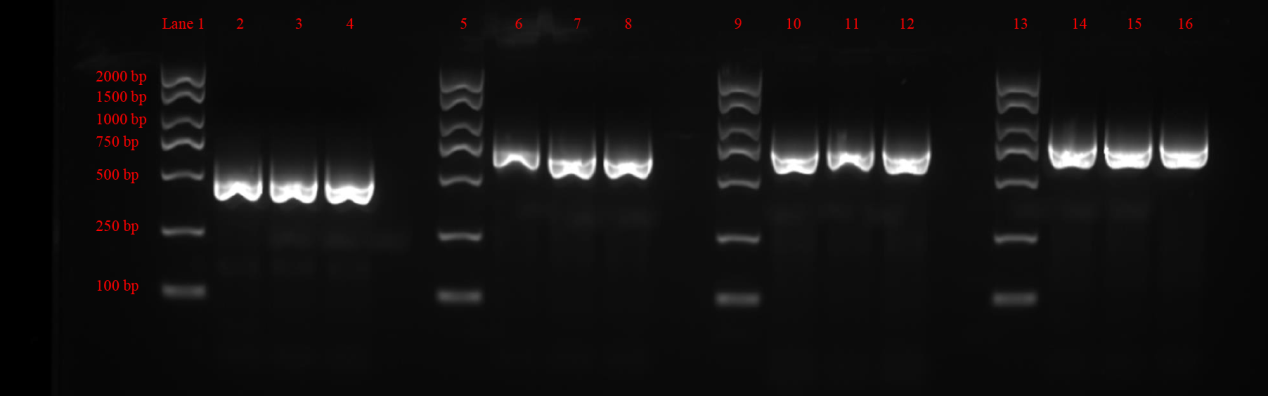


**Figure S2.** Pump *adeABC* systems detected. Lane 1, 5, 9, 13: DNA size marker. Lane 2-4: *adeR* gene, lane 6-8 *adeS* gene, lane 10-12 *adeB* gene, and lane 14-16 *16S rRNA* gene PCR amplification products of MDR-B, MDR-TJ, ATCC 19606, respectively.


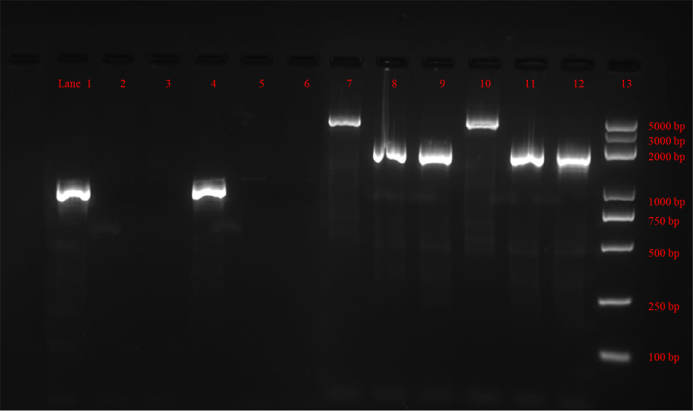


**Figure S3.** Deletion of pump gene *adeB*. Lane1-6: *adeB* gene PCR amplification products. Lane 7-12: UP-DOWN fragment PCR amplification products. Lane 1 and 7 are MDR-B; lane 2, 3, 8, 9 are △*adeB* MDR-B; lane 4 and 10 are MDR-TJ; lane 5, 6, 11, 12 are △*adeB* MDR-TJ, respectively. Lane 13: DNA size marker.
